# Autophagy in pancreatic cancer

**DOI:** 10.1093/jmcb/mjab053

**Published:** 2021-08-30

**Authors:** Congting Guo, Ying Zhao

**Affiliations:** Beijing Key Laboratory of Protein Posttranslational Modifications and Cell Function, Department of Biochemistry and Molecular Biology, School of Basic Medical Sciences, Peking University Health Science Center, Beijing 100191, China; Beijing Key Laboratory of Protein Posttranslational Modifications and Cell Function, Department of Biochemistry and Molecular Biology, School of Basic Medical Sciences, Peking University Health Science Center, Beijing 100191, China

Macroautophagy (hereafter referred to as autophagy) is a conserved eukaryotic process, in which dysfunctional proteins, organelles, and other macromolecules are wrapped in intracellular vesicles called autophagosomes and delivered to lysosomes for degradation (Mizushima et al., 2008; Mizushima and Komatsu, 2011). Autophagy restores cellular energy and recycles cytoplasmic precursors, sustaining cell homeostasis and cell survival (Mizushima et al., 2008; Mizushima and Komatsu, 2011). Elevated autophagy can be detected in both cancer cells and surrounding stromal cells in pancreatic cancer (PC), affecting tumor initiation and progression as well (Yang et al., 2011; Endo et al., 2017). The role of autophagy in PC is highly context-dependent. Here, we illustrate the multifactorial role of autophagy in PC, and the significance of autophagy will be emphasized not only in cancer cells but also in the tumor microenvironment.

## Autophagy through PDAC cell-autonomous mechanisms

### Dual role of autophagy in PDAC

Pancreatic ductal adenocarcinoma (PDAC) is the most prevalent type of PC that counts up to 90% of all diagnosed cases (Adamska et al., 2017). The hypoxic and nutrient-poor tumor microenvironment in PDAC leads to autophagy induction (Sousa et al., 2016). However, the role of autophagy in PDAC is controversial. It has been demonstrated that autophagy plays a dual role in PDAC, both tumor-promoting and tumor-suppressing.

**Figure 1:**
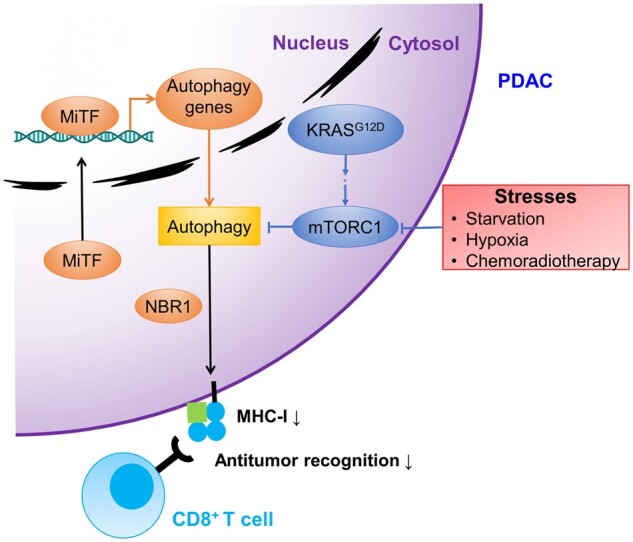
Tumor-promoting role of autophagy in PDAC. Exogenous and endogenous stresses induce autophagy. KRAS signaling is a negative regulating pathway of PDAC autophagy. Activation of autophagy is downstream of microphthalmia transcription factors (MiTF) in the nucleus. Elevated autophagy facilitates PDAC immune escape by degrading MHC-I, and the process is mediated by autophagy cargo receptor neighbor of breast cancer 1 gene (NBR1). Factors promoting autophagy (orange); factors inhibiting autophagy (blue).

Mutation of KRAS, especially with point mutation G12D, is the primary dynamo for PDAC initiation and maintenance (Waters and Der, 2018). Genomic analysis has shown that ∼92% malignant transition from pancreatic ductal intraepithelial neoplasia (PanIN) to advanced PDAC is KRAS-mutated (Bailey et al., 2016). Autophagy is elevated in most KRAS-driven tumors, recycling metabolism precursors to support tumor growth, serving a pro-tumor role (Yang et al., 2011, 2014). Intriguingly, autophagy inhibition sensitizes KRAS-driven PDAC toward ERK inhibitors (Bryant et al., 2019). Blocking the RAF‒MEK‒ERK pathway increases the sensitivity of PDAC to autophagy inhibitors and impedes tumorigenesis as well (Bryant et al., 2019). It is further verified by a clinical practice that used a combination of MEK inhibitor and autophagy inhibitor in a 68-year-old man, who showed little response to standard treatment (Kinsey et al., 2019). After 4-month treatment, half of the tumor load was reduced and partial response was achieved, indicating a promising prospect for clinical application (Kinsey et al., 2019). Thus, the development of PDAC is highly dependent on KRAS and autophagy, again demonstrating the pro-tumor effect of autophagy (Figure 1).

The tumor-promoting role of autophagy in PDAC is further verified in genetic engineering mouse models (GEMMs). Homozygous knockout of the essential autophagy gene Atg5 in mice bearing PDAC significantly prolonged the survival of mice compared to the wild-type and heterozygote Atg5^+/–^ cohorts (Yang et al., 2014). Tumor progression from PanIN, the precursor of PDAC, to invasive PDAC is blocked in mice lack of Atg7 (Rosenfeldt et al., 2013). The underlying mechanisms concerning the pro-tumor role of autophagy are various. Pharmacological suppression of autophagy by chloroquine (CQ) and its derivant hydroxychloroquine (HCQ) results in reactive oxygen species (ROS) upregulation, DNA impairment, and mitochondria dysfunction, ultimately inhibiting cell growth *in vitro* as well as in xenografts (Yang et al., 2011, 2014). In addition, autophagy inhibition leads to decreased amino acid concentration, indicating that autophagy maintains the balance of intracellular amino acid and serves as an independent supplement for PDAC metabolism (Perera et al., 2015). Autophagy also functions in PDAC immune evasion, as it selectively degrades major histocompatibility complex class I (MHC-I), which presents antigen in anti-tumor immunity (Yamamoto et al., 2020). Inhibiting autophagy increases MHC-I levels on the plasma membrane and in total as well, supporting immune presentation and impairing tumor growth (Yamamoto et al., 2020).

Serving as a gatekeeper of cellular homeostasis, the optimal autophagic response is a robust barrier against tumorigenesis by suppressing genomic defects and maintaining cellular metabolism (Galluzzi et al., 2015). Therefore, autophagy can also be a drawback of tumor growth. For example, autophagy-deficient mice with mutant KRAS are at higher risk to develop PanIN (Yang et al., 2014). In addition, complete blockade of autophagy harms cellular metabolism and accelerates tumor growth. p62 (SQSTM1) is a well-known autophagy substrate, which accumulates upon autophagy inhibition (Mathew et al., 2009). It has been shown that aggregates of p62 induce NRF2-mediated MDM2 expression, which accelerates PDAC progression through abrogating p53 or activating the Notch‒p27 axis (Todoric et al., 2017). Consistently, another study performed in RAS-mutated PC and colorectal cancer has demonstrated that autophagy depletion triggers the NF-κB pathway in a p62-dependent manner, which significantly induces epithelial‒mesenchymal transition and promotes tumor invasion (Wang et al., 2019). Besides p62, increased ROS level was also observed in autophagy-deficient cells, leading to Jun kinase activation and overgrowth of Ras^V12^-driven epithelial tissue (Manent et al., 2017). Therefore, autophagy has negative effects on tumor growth, since the accumulation of substrates upon complete blockade of autophagy may serve as pro-tumor regulators.

These findings have defined the different characters of autophagy in PDAC. The double-edged role of autophagy makes it difficult for targeted therapy. Inhibiting autophagy has been proved to be a promising measure in PDAC cell lines and mouse models, while augmented autophagy is correlated with poor patient outcomes (Yang et al., 2011; Perera et al., 2015). Unfortunately, only a few patients show response to autophagy inhibitors in clinical trials, and adding HCQ in chemotherapy does not improve 1-year survival or overall survival (Karasic et al., 2019). Thus, it is plausible that the addition of autophagy inhibitors alone is insufficient to halt PDAC development. Given the inspiring research using HCQ and inhibitor of KRAS signaling to treat PDAC (Kinsey et al., 2019), combination therapy may provide a potential strategy to cure PC. Not only PDAC, other KRAS-mutant cancers, such as colorectal cancer are also sensitive to co-target of autophagy essential gene Atg7 and RAF kinases, the subset of KRAS signaling (Lee et al., 2019). Moreover, since most autophagy inhibitors in clinical trials are given systemically, more specific and targeted therapies need to be developed.

### The role of autophagy in PC is related to genetic status

**Figure 2:**
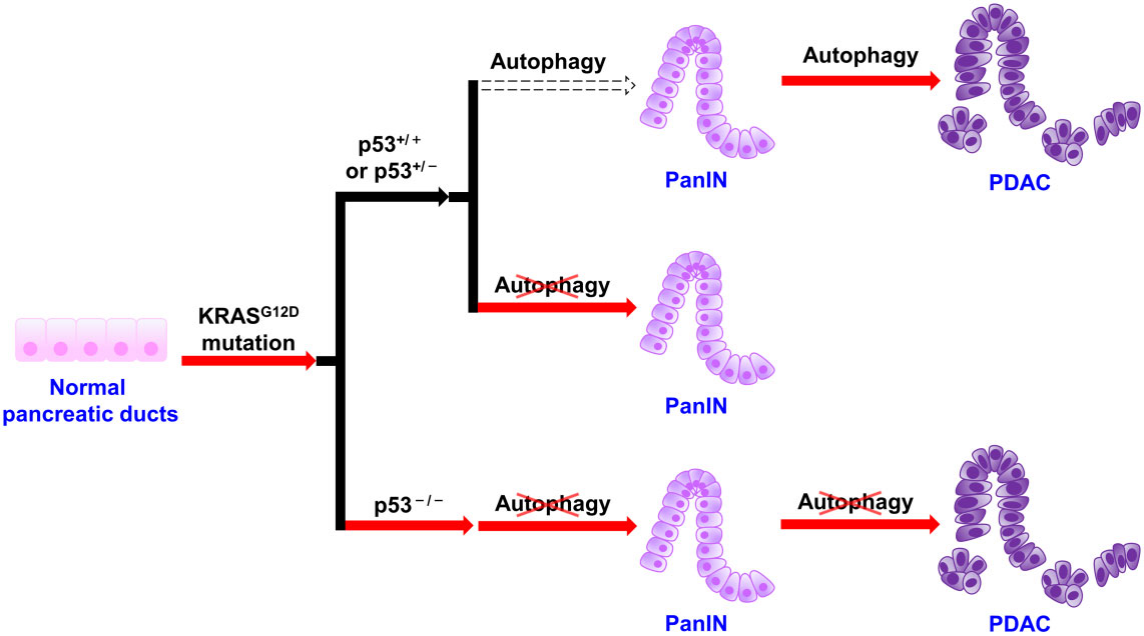
The role of autophagy in PC is related to genetic status. Initially, KRAS mutation drives normal pancreatic ducts to tumorigenesis. Under p53^+/+^ or p53^+/–^ status, autophagy impedes cell malignant transformation, while supporting PDAC progression. Autophagy-deficient cells with mutant KRAS are more likely to develop into benign PanIN but rarely turn into PDAC. Homozygous knockout of p53 breaks the block of tumor progression by autophagy deficiency and accelerates tumor growth. Factors promoting tumorigenesis (red arrow); factors blocking tumorigenesis (dotted arrow).

Mutation of tumor suppressor gene p53 is the second leading genomic change in PDAC that aggregates in 78% of cases (Bailey et al., 2016). Instead of protecting mice from tumor progression, autophagy deficiency actually accelerates the transformation from PanIN to PDAC in mice that harbor both KRAS mutation and homozygous deletion of p53 (Rosenfeldt et al., 2013). Therefore, p53 plays a critical role in blocking tumor progression in the context of autophagy deprivation (Rosenfeldt et al., 2013). Nevertheless, the coexistence of mutant KRAS and homozygous loss of p53 may not occur in human PDAC, in which stochastic loss of heterozygosity of p53 is more common (Yang et al., 2014). Intriguingly, GEMMs and patient-derived xenografts are both sensitive to CQ treatment, indicating the clinical utility of autophagy inhibitor is irrespective of p53 status (Yang et al., 2014; Figure 2). Thus, it is important to note that the role of autophagy in PC is related to genetic status. Personalized treatment concerning genetic status would be of interest to explore.

## Autophagy through non-autonomous mechanisms

**Figure 3:**
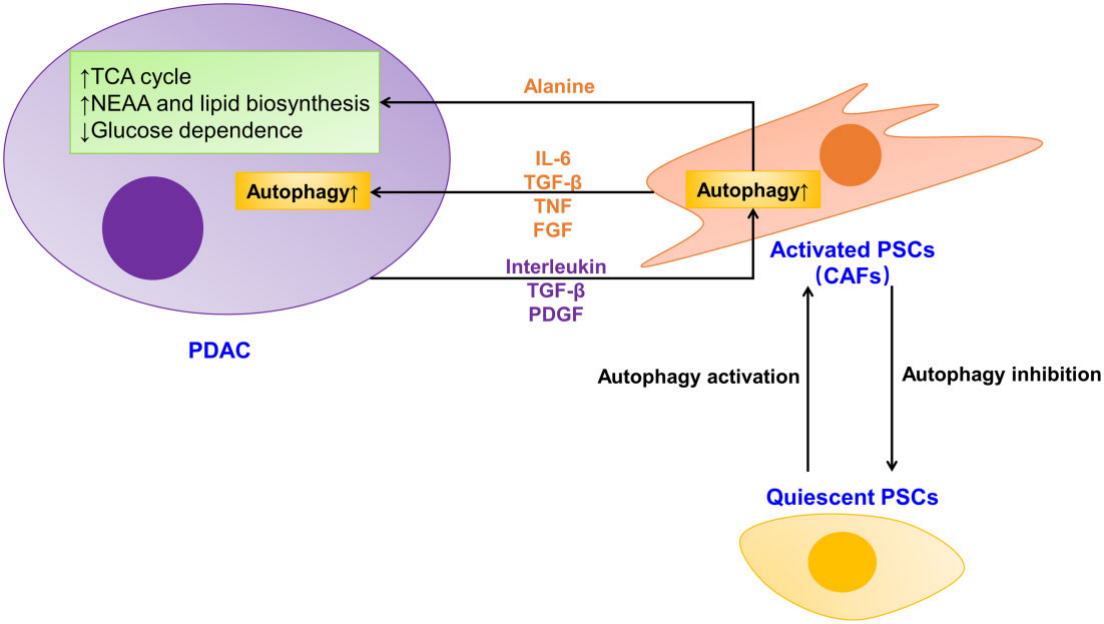
Autophagy through non-cell-autonomous mechanisms. Autophagy stimulates PSCs from quiescent state to activated state, which secretes alanine to support PDAC metabolism. The crosstalk between PDAC and PSCs involves multiple factors and promotes autophagy in both cells.

The microenvironment of tumors has long been considered a contributor to malignant development (Katheder et al., 2017). Pancreatic stellate cells (PSCs) are the most critical stroma cells that interact with PDAC and play an important role in tumor growth, metastasis, and chemo-/radio-resistance (Endo et al., 2017). There is a transition from quiescent PSCs to cancer-associated fibroblasts (CAFs), which are in a hyper-activated myofibroblast-like state and secrete a variety of extracellular matrix proteins and factors to support tumor metabolism (Endo et al., 2017). Autophagy is required for the activation process of PSCs (Endo et al., 2017). Consistently, autophagy inhibition in CAFs results in smaller tumor volume and less liver metastases, leading to longer postoperative survival (Endo et al., 2017). Thus, converting CAFs back to quiescent PSCs by suppressing autophagy may be a possible way to hamper PDAC metabolism (Figure 3).

Several studies have demonstrated that there is an autophagy-related crosstalk between PDAC and PSCs. PDAC secretes multiple cytokines, such as platelet-derived growth factor, transforming growth factor-β (TGF-β), and interleukin (IL), acting on PSCs to stimulate autophagy (Jones et al., 2008; Endo et al., 2017). Accordingly, activated PSCs also secrete fibroblast growth factor, TGF-β, and IL-6 to promote PDAC cell proliferation and metastasis in an autophagy-dependent manner (Endo et al., 2017). Besides cytokines, activated PSCs also provided amino acids (e.g. alanine) to support tumor metabolism (Sousa et al., 2016). As we know, PDAC presents a fierce desmoplastic response and dysregulated metabolism, which leads to an austere tumor microenvironment that is lack of nutrition (Feig et al., 2012; Kamphorst et al., 2015; Sousa et al., 2016). PDAC stimulates non-cell-autonomous autophagy in PSCs, which secret alanine as an alteration carbon source that subsequently fuels tricarboxylic acid cycle by converting to pyruvate in PDAC, thus attenuating the dependence of PDAC on glucose and serum-derived nutrients (Sousa et al., 2016). In addition, alanine facilitates the biosynthesis of other non-essential amino acids and lipids to support PDAC cell proliferation (Sousa et al., 2016). Thus, we can conclude that the tumor-growth-promoting role of PSCs is mediated by autophagy, and inhibiting autophagy in activated PSCs (CAFs) may be a feasible way to cut off the supply to PDAC and impair tumor growth.

Not only does autophagy connects PSCs and PDAC in the tumor microenvironment, but the role of systematical autophagy has also been emphasized in PDAC. Atg4B is a deconjugating enzyme and functions in the formation of autophagosome membrane (Mizushima et al., 2011). A GEMM with a mutation of Atg4B was used to test the role of autophagy in PDAC (Yang et al., 2018). Not surprisingly, mice bearing two copies of mutated Atg4B, which stands for whole-body abolishment of autophagy, show the tumor growth regression and prolonged survival even in PDAC that has already formed, reconfirming the critical role of macroenvironmental autophagy in PDAC development (Yang et al., 2018).

The importance of non-cell-autonomous autophagy in the tumor is not confined to PC. *Drosophila* Ras^V12^scrib^−/−^ eye imaginal disc tumor cells secret IL-6 through tumor necrosis factor (TNF)‒JNK‒Fos pathway in response to TNF stimulation (Katheder et al., 2017). IL-6 in turn induces autophagy in both tumor microenvironment and distant tissues, recycling nutrients, such as amino acids to facilitate tumor metabolism (Katheder et al., 2017). Another research performed in mouse melanoma shows that autophagy maintains tumor growth by circulating arginine (Poillet-Perez et al., 2018). Upon systematically or liver-targeted autophagy depletion, serum arginine-degrading enzyme arginase I levels significantly rise and degrades arginine into ornithine (Poillet-Perez et al., 2018). Given that arginine is a semi-essential amino acid, which relies largely on exogenous uptake, blockade of autophagy would cut the supply of arginine in melanoma as well as multiple other human cancers and thus is a potential therapeutic strategy upsetting tumor homeostasis (Poillet-Perez et al., 2018).

## Conclusion

Over the past few years, our understanding of autophagy and PC has increased strikingly. However, there are still many mechanisms remaining to be uncovered. The majority of existing studies focus on cell lines or GEMMs, and practices in patients need further exploration. Future studies should probe into the contribution of autophagy to PDAC and tumor micro-/macroenvironment, and the difference between murine and human PDAC needs clarification. This would help us better harness autophagy in human PC and guide the development of other anti-cancer therapies as well.

*[This work was supported by grants from the National Key R&D Program of China (2017YFA0503900) and the National Natural Science Foundation of China (81472581, 81672712, 81874145, and 81621063).]*
